# Upregulating Noxa by ER Stress, Celastrol Exerts Synergistic Anti-Cancer Activity in Combination with ABT-737 in Human Hepatocellular Carcinoma Cells

**DOI:** 10.1371/journal.pone.0052333

**Published:** 2012-12-20

**Authors:** Hong Zhu, Wei Yang, Ling-juan He, Wan-jing Ding, Lin Zheng, Si-da Liao, Ping Huang, Wei Lu, Qiao-jun He, Bo Yang

**Affiliations:** 1 Institute of Pharmacology and Toxicology, College of Pharmaceutical Sciences, Zhejiang University, Hangzhou, China; 2 Tongde Hospital of Zhejiang Province, Zhejiang Academy of Traditional Chinese Medicine, Hangzhou, China; 3 Institute of Medicinal Chemistry, Department of Chemistry, East China Normal University, Shanghai, China; Osaka University Graduate School of Medicine, Japan

## Abstract

The human hepatocellular carcinoma (HCC) represents biologically aggressive and chemo-resistant cancers. Owing to the low affinity with the apoptotic factor Mcl-1, the BH3 mimetic drug ABT-737 failed to exert potent cancer-killing activities in variety of cancer models including HCC. The current study demonstrated that combining ABT-737 and Celastrol synergistically suppressed HCC cell proliferation, and induced apoptosis which was accompanied with the activation of caspase cascade and release of cytochrome c from mitochondria. Further study revealed that the enhanced Noxa caused by Celastrol was the key factor for the synergy, since small interfering RNA-mediated knockdown of Noxa expression in HCC cells resulted in decreased apoptosis and attenuated anti-proliferative effects of the combination. In addition, our study unraveled that, upon Celastrol exposure, the activation of endoplasmic reticulum (ER) stress, specifically, the eIF2α-ATF4 pathway played indispensable roles in the activation of Noxa, which was validated by the observation that depletion of ATF4 significantly abrogated the Noxa elevation by Celastrol. Our findings highlight a novel signaling pathway through which Celastrol increase Noxa expression, and suggest the potential use of ATF4-mediated regulation of Noxa as a promising strategy to improve the anti-cancer activities of ABT-737.

## Introduction

ABT-737 is a potent small-molecule inhibitor, which targets the Bcl-2-regulated apoptosis pathway, serving as a Bad-like BH3 mimetic. It selectively bounds to Bcl-2, Bcl-XL and Bcl-w but not Mcl-1 and Bfl-1/A1. In preclinical studies, ABT-737 has shown single-agent activity against various leukemia [1], lymphoma [2], and small cell lung cancer [3]. While ABT-737 has been shown to be a promising therapeutic agent, it is unlikely to be effective as a single agent in solid tumors resulted from its low affinity with Mcl-1 and high level of Mcl-1 expression in cancer cells [4]–[7]. This makes the exploration of combination strategies crucial for improving current treatment of ABT-737 against cancer, of which the hot issue is to combine ABT-737 with other drugs which have the ability to modulate Mcl-1. In our previous studies, we found that gemcitabine could enhance ubiquitination and the subsequent degradation of Mcl-1, therefore exhibited synergistic cytotoxicity with ABT-737 in multiple types of cancer cells [6]. Similarly, GDC-0941-promoting degradation of Mcl-1 was also responsible for its synergistic killing of breast cancer cells with ABT-737 [5]. Therefore, the modification on Mcl-1 expression would trigger sensitization of ABT-737 in cancel cells.

The BH3-only protein Noxa is able to selectively interact with Mcl-1, then release Bak or Bax from Mcl-1 to activate the mitochondrial apoptosis pathway or target it for proteasomal degradation [5]–[8]. Due to its typical characteristic, Noxa has been highlighted as an effective factor to reverse the resistance to ABT-737 which is caused by Mcl-1. Lucas KM et al indicated that overexpression of Noxa strongly overcame ABT-737 resistance in melanoma cells [9]. Besides, Noxa-inducing agents have also been reported to sensitize cancer cells to ABT-737, including Bortezomib [10], Fludarabine [11], Oxaliplatin [12], etc. Recently, Dai Y et al demonstrated that Celastrol, a natural extract with potent anti-cancer capabilities, could lead to induction of Noxa and cleavage of Mcl-1 [13], which attracted our attention to examine the effects when combine this agent with ABT-737, whose anti-cancer activities were closely related to Mcl-1.

Celastrol is a pharmacologically active compound originally identified from traditional Chinese medicine Thunder of God Vine root extracts, and has been used as a natural remedy for inflammatory conditions and anticancer treatment for years [14]. As a HSP90 inhibitor, Celastrol disrupted HSP90-Cdc37 interaction against pancreatic cancer cells [15], [16], and imposed influence on ER-stress response [17]. In addition, Celastrol could induce apoptosis by activating Noxa and modulating Mcl-1 [13], with detailed mechanisms unknown, and the potential application remain elusive.

In this study, we investigated the potentially synergistic abilities of ABT-737 in combination with Celastrol in human hepatocellular carcinoma cell lines, in which mostly harbor high level of Mcl-1 protein expression [18]. The combination index (CI) values of the anti-proliferative capabilities on two human liver cancer cell lines Bel-7402 and HepG2 were less than 0.7, indicating the synergism of the combination of ABT-737 and Celastrol. Furthermore, Celastrol greatly potentiated ABT-737-mediated apoptosis in Bel-7402 and HepG2 cells by stimulating Noxa expression and its interaction with Mcl-1, which was dependent on the induction of ER stress response, specifically, the activation of ATF4. In general, our study firstly determined the synergistic effects of ABT-737 plus Celastrol in human hepatocellular carcinoma cells, opening the opportunity of combining these two agents as potent therapeutic combination, and implying that the activation of ER stress which lead to the manipulation on Noxa might served as a effective strategy to inhibit Mcl-1 and thus to increase the anti-cancer activities of ABT-737.

**Figure 1:**
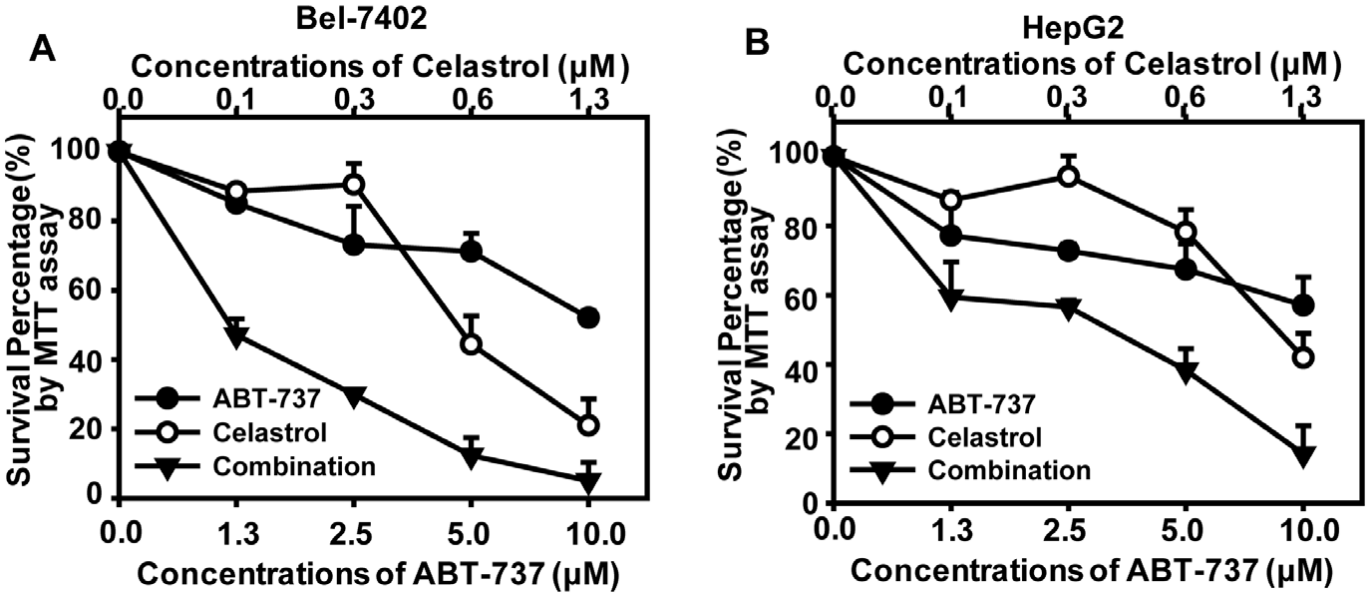
Combination cytotoxicity of ABT-737 and Celastrol. MTT assays were used to examine the cell proliferation inhibitory activities in human liver cancer Bel-7402 (**A**) and HepG2 (**B**) cells. Cells in 96-well plates were exposed to serial concentrations of ABT-737, Celastrol or the constant-ratio mixture of these two for 72 h. The concentrations applied were 1.25–10 µM for ABT-737, 0.16–1.25 µM for Celastrol. Concentration–response curves of two cell lines to ABT-737, Celastrol and the combination were presented. Three independent experiments were performed, and standard deviation was represented as error bars. The CI values were demonstrated in Table 1.

**Table 1:** CI Values of ABT-737 at Concentrations Applied in Combination with Celastrol in human liver cancer Bel-7402 and HepG2 cells.

Bel-7402			HepG2		
ABT-737 (µM)	Celastrol (µM)	CI	ABT-737 (µM)	Celastrol (µM)	CI
1.3	0.16	0.26	1.3	0.16	0.28
2.5	0.31	0.27	2.5	0.31	0.48
5.0	0.63	0.21	5.0	0.63	0.37
10.0	1.25	0.20	10.0	1.25	0.21
Mean	–	0.24	Mean	–	0.34

A CI less than 0.90 indicates synergism; 0.10, very strong synergism; 0.10–0.30, strong synergism; 0.30–0.70, synergism; 0.70–0.85, moderate synergism; 0.85–0.9, slight synergism; 0.90–1.10, additive; and more than 1.10, antagonism.

## Materials and Methods

### 1. Chemicals and Reagents

ABT-737 was purchased from Selleck Chemicals (Houston, TX). Celastrol was synthesized by Professor Wei Lu (East China Normal University) with purity greater than 99%. Both ABT-737 and Celastrol were dissolved in dimethylsulfoxide at the stock concentration of 20 mM (DMSO). The primary antibodies against PARP, pro-caspase-3, Bax, Bim, Bcl-xL, ubiquitin, Actin and HRP-labeled secondary anti-goat, anti-mouse and anti-rabbit antibodies were purchased from Santa Cruz Biotechnology (Santa Cruz, CA); the primary antibodies against cleaved-caspase-3, Puma, cytochrome c, Bcl-2, Mcl-1, ATF4, Chop, p-eIF2α (Ser51), eIF2α, HSP70, p-ERK, ERK, and CDK4 was purchased from Cell Signaling Technology (Danvers, MA); the primary antibodies against Noxa was purchased from Calbiochem (Darmstadt, Germany).

### 2. Cell Culture

Human hepatocellular carcinoma cell lines Bel-7402 and HepG2 were purchased from Shanghai Institute of Biochemistry and Cell Biology (Shanghai, China) and maintained in a 5% CO_2_ atmosphere at 37°C. Bel-7402 cells were cultured in RPMI-1640 medium supplemented with 10% fetal bovine serum and HepG2 cells in Dulbecco’s modified Eagle’s medium supplemented with 10% fetal bovine serum.

**Figure 2:**
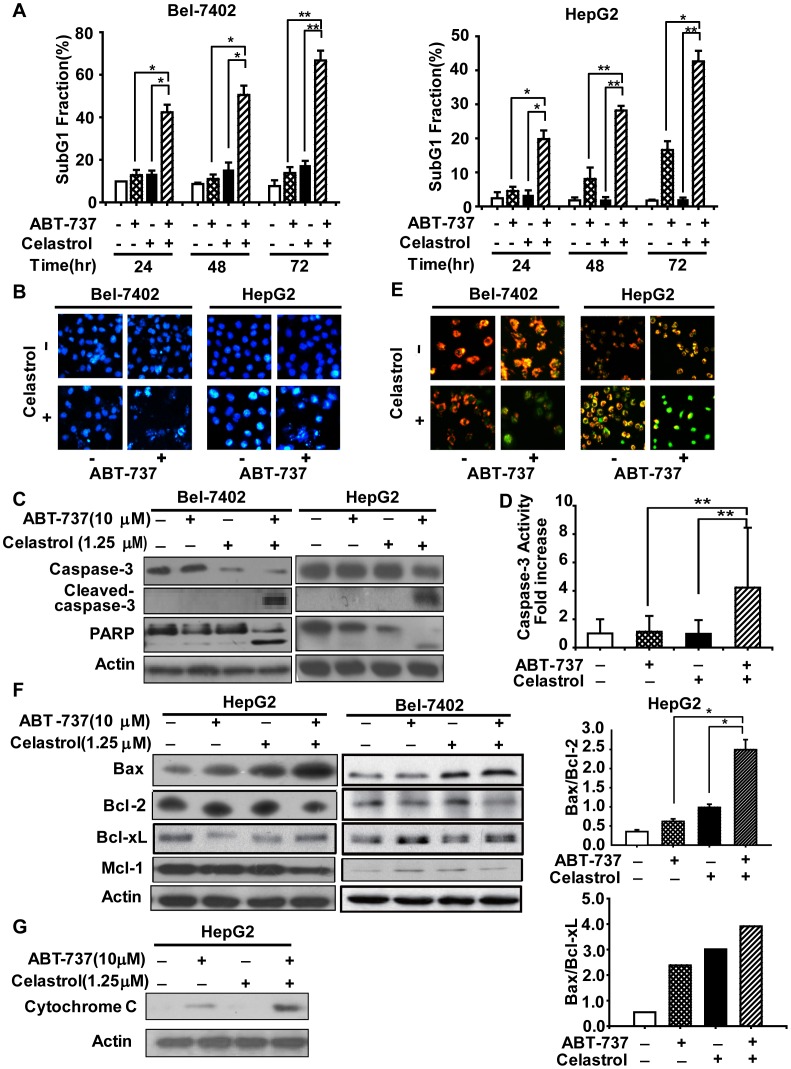
ABT-737 synergized with Celastrol to trigger apoptosis. **A.** Cells were treated with 10 µM ABT-737, 1.25 µM Celastrol and the combination for 24, 48, 72 hours and apoptosis was tested by propidium iodide staining of lysed cell nuclei, analyzed by Flow Cytometry. **B.** After treatment 10 µM ABT-737, 1.25 µM Celastrol and the combination for 72 hours, cells were stained with DAPI and observed by Leica DMI 400B Xuorescence microscope. Cells were treated with 10 µM ABT-737, 1.25 µM Celastrol and the combination for 24 h; **C.** lysates were harvested and immunobloted with caspase-3, cleaved caspase-3 and PARP antibodies; **D.** Caspase-3 activity were determined using the specific substrate Ac-DEVDPNA; HepG2 cells were treated with 10 µM ABT-737, 1.25 µM Celastrol and the combination for 24 h. **E.** Bel-7402 and HepG2 cells were stained with JC-1, then observed by Leica DMI 400B Xuorescence microscope; **F.** protein levels of Bax, Bcl-2, Bcl-xL and Mcl-1 in HepG2 and Bel-7402 cells were detected by western blotting analysis. The density of the protein band was determined by using Bio-Rad Quantity One imaging software; **G.** the separation of cytosol was accomplished. The cytosol samples were analyzed, and the release of cytochrome c was evaluated by western blotting analysis. The results were similar in at least three independent experiments. *, *p*<0.05; **, *p*<0.01.

### 3. Cell Survival Assay

Cell survival was assessed by MTT cell viability assay. Briefly, exponentially growing Bel-7402 and HepG2 cells were seeded into 96-well plates and cultured overnight in a 5% CO_2_ atmosphere at 37°C before treatment of exposed to DMSO vehicle, serial concentrations of ABT-737, Celastrol or the combination for 72 h. Then, cells were incubated with MTT (5 mg/ml, 20 µl/well) for 4 h and the formazan granules generated by live cells were dissolved in DMSO. The absorbance at 570 nm was measured using a multiscan spectrum (Thermo Electron Corporation Marietta, OH). Assays were performed in triplicate in three independent experiments.

### 4. Analysis of Apoptosis by DAPI Staining

Exponentially growing Bel-7402 and HepG2 cells were seeded into 96-well plates and cultured overnight in a 5% CO_2_ atmosphere at 37°C before treatment of DMSO vehicle, serial concentrations of ABT-737, Celastrol or the combination for 72 h. Harvested cells were washed once with PBS, incubated with 0.1% Triton and 0.1% DAPI at room temperature for 3 min, then washed twice with PBS and imaged with Leica DMI 400B fluorescence microscope.

**Figure 3:**
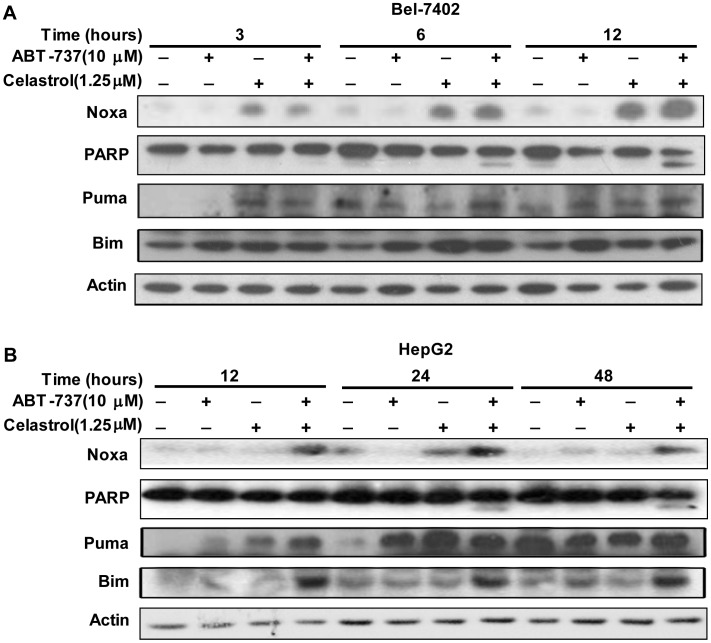
Celastrol increased NOXA protein levels earlier than cleaved-PARP in Bel-7402 and HepG2 cells. **A.** Bel-7402 cells were treated with 10 µM ABT-737, 1.25 µM Celastrol and the combination for 3, 6, 12 h. **B.** HepG2 cells were treated with10 µM ABT-737, 1.25 µM Celastrol for 12, 24, 48 h. Then lysates were harvested and immunobloted with NOXA, Bim, PUMA and PARP antibodies.

### 5. Analysis of Apoptosis by PI Staining

Exponentially growing Bel-7402 and HepG2 cells were seeded and cultured overnight in a 5% CO_2_ atmosphere at 37°C before treatment of DMSO vehicle, serial concentrations of ABT-737, Celastrol or the combination for 24 h, 48 h and 72 h. Harvested cells were washed once with PBS and fixed with cold 70% ethanol at −20°C for at least 2 h. The fixed cells were washed twice with PBS and resuspended in PBS containing 40 µg/mL RNase A at 37°C for 30 min and 10 µg/mL propidium iodide in dark at room temperature for 30 min. Flow cytometry was performed on FACScan (BD Biosciences, San Jose, CA).

### 6. Determination of Mitochondrial Membrane Depolarization

Exponentially growing Bel-7402 and HepG2 cells were seeded and cultured overnight in a 5% CO_2_ atmosphere at 37°C before treatment of DMSO vehicle, serial concentrations of ABT-737, Celastrol or the combination for 24 h. Harvested cells were washed once with PBS and resuspended in PBS containing 10 µg/mL JC-1 (5,5′,6,6′-Tetrachloro-1,1′,3,3′- tetraethylbenzimidazolyl-arbocyanine iodide). After incubationat 37°C for 20 min, cells were washed twice with PBS, then imaged with Leica DMI 400B fluorescence microscope or analyzed by flow cytometry.

**Figure 4:**
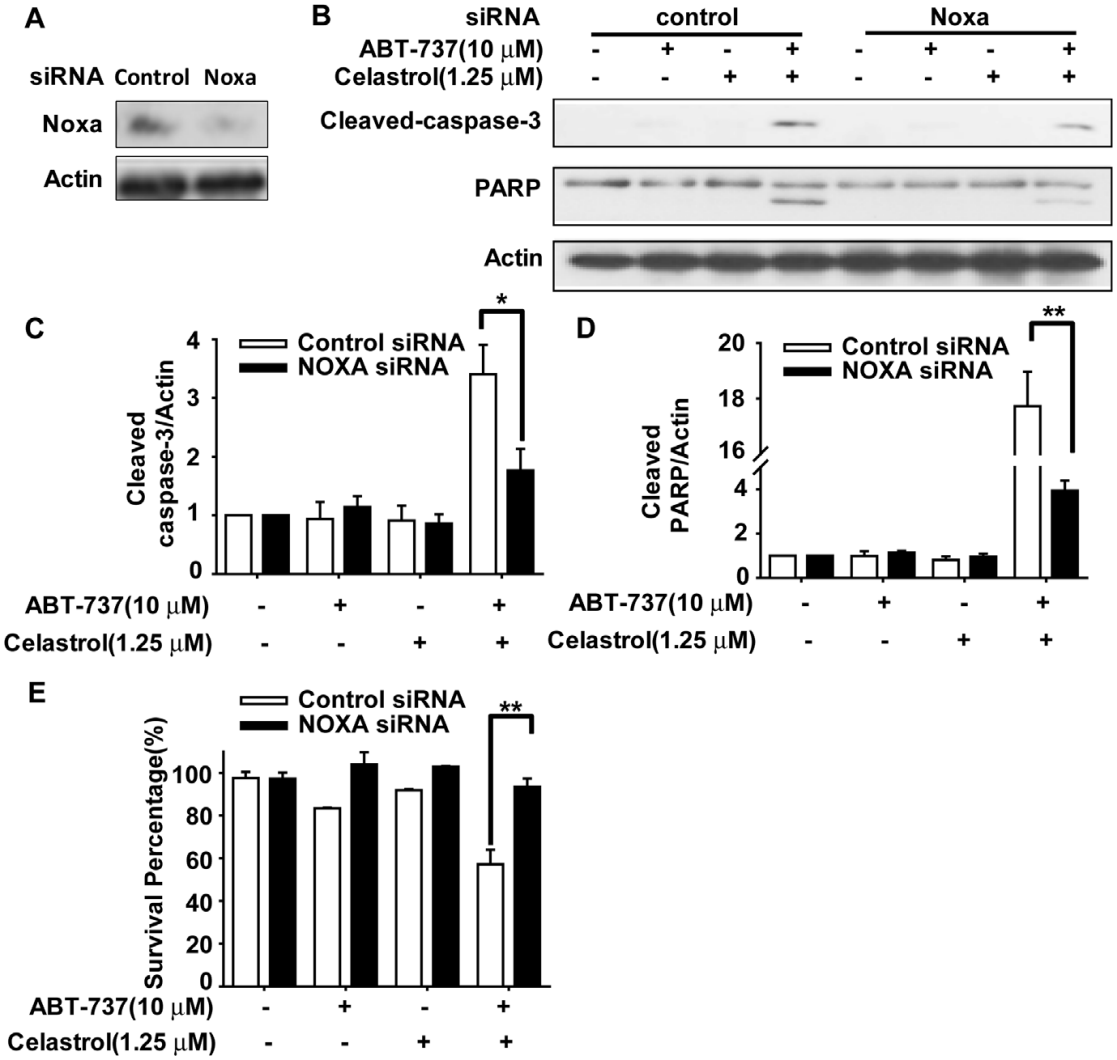
Noxa was required in potent augmentation of ABT-737-killing by Celastrol. HepG2 cells were transfected with NOXA siRNA according to manufacturer’s recommendations. Forty-eight hours after transfection, cells were treated with10 µM ABT-737, 1.25 µM Celastrol and the combination for 48 h. **A.** Lysates were harvested and immunobloted with NOXA antibody. **B.** Lysates were harvested and immunobloted with cleaved caspase-3 and PARP antibodies. **C.** The ratios of cleaved-caspase 3/β-Actin. **D.** The ratios of cleaved-PARP/Actin. The density of the protein band was determined by using Bio-Rad Quantity One imaging software. **E.** The cell survival fractions were detected by MTT assay. *, *p*<0.05; **, *p*<0.01.

### 7. Western Blot Analysis

Bel-7402 and HepG2 cells were harvested, washed once with PBS, and resuspended in lysis buffer (50 mM Tris-HCl, 150 mM NaCl, 1 mM EDTA, 0.1% SDS, 0.5% deoxycholic acid, 0.02% sodium azide, 1% NP-40, 2.0 µg/ml aprotinin, 1 mM phenylmethylsulfonylfluoride) on ice for 30 min. The lysates were centrifuged at 13,000 rpm for 30 min at 4°C. The concentrations of the total lysate protein were detected by standard Bradford assay (Bio-Rad, San Diego, CA). For Western blot analysis, 40–100 µg of proteins were electrophoresed by SDS-PAGE, transferred to nitrocellulose membrane (Pierce Chemical) and probed with primary antibodies followed by horseradish peroxidase-coupled secondary antibodies at 1∶5000 dilution. Proteins were visualized using enhanced chemiluminescence (ECL)-plus kit from Amersham Biosciences (UK).

### 8. Measurement of Caspase-3 Activity

The activity of caspase-3 was measured by cleaving selective substrate acetyl-Asp-Glu-Val-Asp P-nitroanilide (Ac-DEVDPNA) (Beyotime institute of biotechnology). Cells were lysed and protein concentration of supernatants was measured by Bradford's method and equal amounts of proteins (10 µL) were incubated in a total volume of 100 µL comprised of 80 µL detection buffer. The reaction was started by addition of caspase-3 substrates Ac-DEVD-PNA (10 µL). After incubation for 60 min at 37°C, cleavage of the substrate was detected using a multiscan spectrum (Thermo Electron Corporation Marietta, OH) at wavelength of 405 nm. Activities of caspase-3 were expressed as changes in DEVDase activity.

### 9. Detection of Release of Cytochrome c

Cells were harvested, washed once with PBS, and resuspended in an extraction buffer [20 mmol/L HEPES (pH 7.5), 1.5 mmol/L MgCl_2_, 10 mmol/L KCl, 1 mmol/L EGTA, 1 mmol/L EDTA, 250 mmol/L sucrose, 0.1 mmol/L phenylmethylsulfonyl fluoride and 1 mmol/L DTT], and homogenized using a microhomogenizer. The homogenates were centrifuged at 750×g for 10 min at 4°C. Then the supernatants were centrifuged at 10,000×g for 15 min at 4°C, and the remaining supernatants were regarded as cytosol fraction. After Western blot analysis, anti-cytochrome c antibodies (Cell Signaling Technology) were used to detected the mitochondrial release of cytochrome c.

**Figure 5:**
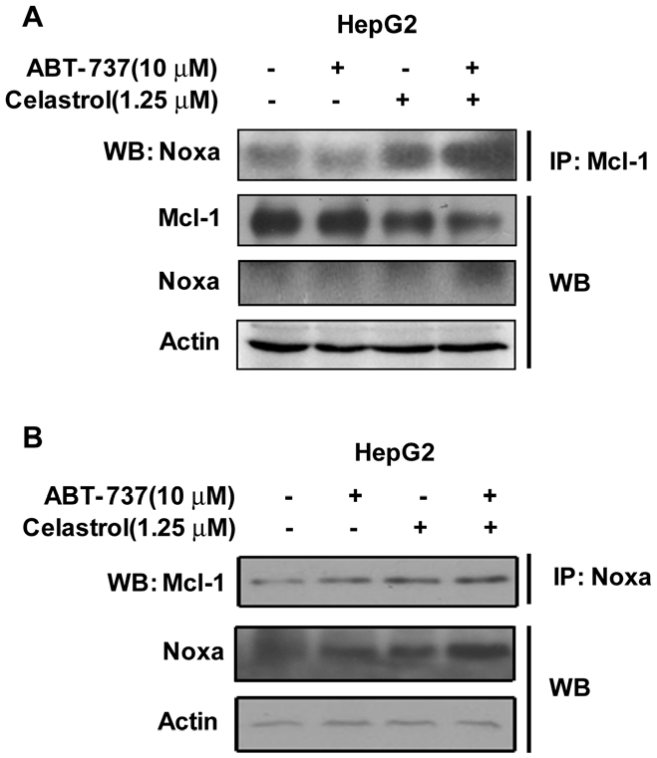
Binding between Mcl-1 and NOXA in ABT-737 and Celastrol-treated HepG2 cells. After treatment of 10 µM ABT-737, 1.25 µM Celastrol and the combination for 24 h, cellular extracts were immunoprecipitated with Mcl-1 (**A**) or Noxa (**B**) antibodies. Immunoprecipitates were subjected to SDS-PAGE and probed with Noxa (**A**) or Mcl-1 (**B**) antibodies. Lysates were harvested and immunobloted with Mcl-1, Noxa and Actin antibodies.

**Figure 6:**
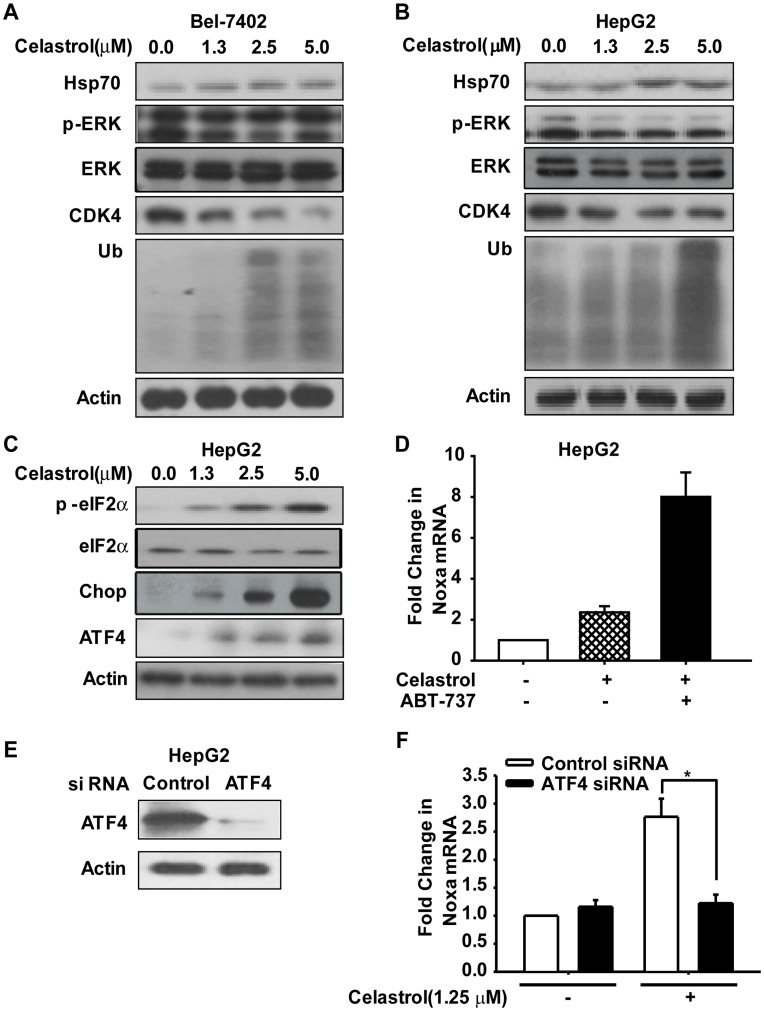
Celastrol-caused Hsp90 inhibition and ER-Stress response let to increase of Noxa. **A and B.** Bel-7402 and HepG2 cells were treated 1.25, 2.5, 5 µM Celastrol for 3 h, lysates were harvested and immunobloted with Hsp70, p-ERK, CDK4 and UB antibodies. **C.** HepG2 cells were treated 1.25, 2.5, 5 µM Celastrol for 3 h, lysates were harvested and immunobloted with p-eIF2a and ATF4. **D.** HepG2 cells were treated with 1.25 µM Celastrol or plus 10 µM ABT-737 for 12 h. Noxa mRNA levels were determined by real-time RT-PCR relative to GAPDH as an internal control. **E.** HepG2 cells were transfected with ATF4 siRNA according to manufacturer’s recommendations. Forty-eight hours after transfection, lysates were harvested and immunobloted with ATF4 antibody. **F.** Cells were treated with 1.25 µM Celastrol for 24 h. Noxa mRNA levels were determined by real-time RT-PCR relative to GAPDH as an internal control. The results were similar in at least three independent experiments. *, *p*<0.05.

### 10. Reverse Transcription PCR Assay

Total RNA was prepared using the trizol, precipitated by isopropyl alcohol and rinsed with 70% ethanol. Single-strand cDNA was prepared from the purified RNA using oligo(dT) priming (Thermoscript RT kit; Invitrogen), followed by SYBR-Green real-time PCR (Qiagen). The primers were as follows: Noxa, 5′-ATGAATGCACCTTCACATTCCTCT-3′,5′-TCCAGCAGAGCTGGAAGTCGAGTGT-3′ [19];GAPDH, 5′-TGCACCACCAACTGCTTAGC-3′, 5′-GGCATGGACTGTGGTCATGAG-3′ [20]. Relative expression levels of the target genes were normalized with the control gene GAPDH. For the detection of Xbp1 splicing, the conditions used for reverse transcription-PCR were as follows: 10 min at 25°C, 60 min at 42°C and 15 min at 72°C. The cDNA was subjected to PCR amplification using the following forward and reverse primer: Xbp1, forward primer: 5′-CCTTG TAGTTGAGAACCAGG-3′ and reverse primer: 5′-GGGGCTTGGTATATATGTG G-3′ [21]. PCR products were separated on 1.0% agarose gel and visualized by ethidium bromide staining. Gels were photographed using a Gel DOC 2000 image analyzer (Bio-Rad, Hercules, CA, USA).

### 11. Silencing of Gene Expression with Small Interfering RNA(siRNA)

Exponentially growing cells were seeded in 6-well plates (4×10^4^/well) and cultured overnight in a 5% CO_2_ atmosphere at 37°C. Then the medium was replaced with Opti-MEM I Reduced Serum Media (GIBCO) containing 20.0 nM Mcl-1 or ATF4 siRNA (GenePharma, China) and oligofectamine reagent (Invitrogen Corporation) according to manufacturer’s recommendations. The sense sequences of siRNA were as follows: NOXA: 5′-UCAGUCUACUGAUUUACUGG-3′ [22]; ATF4: 5′-GCGUAGUUCGCUAAGGUGAdTdT-3′ [23]. Forty-eight hours after transfection, cells were harvested or treated with DMSO vehicle, serial concentrations of ABT-737, Celastrol or the combination.

### 12. Immunoprecipitation

Cells were harvested and resuspended in universal lysis/immunoprecipitation buffer (50 mM Tris-HCl, 150 mM NaCl, 2 mM EDTA, 2 mM EGTA, 25 mM NaF, 25 mM β-glycerophosphate, pH 7.5, 0.1 mM sodium orthovanadate, 0.1 mM PMSF, 5 µg/ml leupeptin, 0.2% Triton X-100, 0.5% Nonidet P-40) on ice for 30 min. The lysates were centrifuged at 13,000 rpm for 30 min at 4°C. 300 µg cellular proteins were precleared by adding 1 µg of normal immunoglobulin G together with 50 µl of appropriate protein A+G-agarose conjugate (Santa Cruz) for 1 h at 4°C. The immunoprecipitations were performed with the appropriate primary antibody overnight at 4°C. Complexes bound to the protein A+G-agarose conjugate were washed seven times with universal lysis/immunoprecipitation buffer and fractioned by SDS-PAGE. The Western blot analysis was then performed.

### 13. Statistical Analysis

Combination index (CI) is well accepted for quantifying drug synergism based on the multiple drug effect equation of Chou-Talalay [24]. In our study, CIs for each concentration of ABT-737, Celastrol and the combination in cell survival assays were calculated by Calcusyn Software (Biosoft, Cambridge, United Kingdom). A CI lower than 0.9 indicates synergism; a CI of 0.9 to 1.10 indicates additive; and a CI higher than 1.10 indicates antagonism.

Differences in sub-G1 population, caspase-3 activity and Bax/Bcl-2 ratiofor each two groups (combination *vs.* ABT-737; combination *vs.* Celastrol), and differences in cleaved caspase-3/actin, cleaved PARP/actin, and survival percentage of combination group in Noxa silenced cells *vs.* control siRNA cells, fold change in Noxa mRNA of Celastrol-treated cells in ATF4 siRNA cells *vs.* control siRNA cells, were evaluated by unpaired two-sided Student’s t-test and indicated with ** for p<0.01 and * for p<0.05. For each analysis, three independent experiments were conducted to obtain the data.

## Results

### 1 Cytotoxicity of the ABT-737 and Celastrol Combination in Human Hepatocellular Carcinoma Cell Lines

Firstly, by using MTT assay we evaluated the cytotoxicity of ABT-737, Celastrol and the combination at the indicated concentrations for 72 h in two HCC cell lines Bel-7402 and HepG2. Corresponding survival fraction curves were shown in Fig. 1. In comparison with ABT-737 or Celastrol alone, the combination of ABT-737 and Celastrol exerted more significant anti-proliferation effects in both Bel-7402 and HepG2. CI values were calculated by Calcusyn Software at the fixed-ratio concentrations of ABT-737 and Celastrol (Table 1). Synergy (CI<0.70) or strong synergy (CI<0.30) was observed in both tested cancer cell lines.

### 2 ABT-737 Synergized with Celastrol to Trigger Apoptosis

#### 2.1 ABT-737 plus celastrol induced enhanced apoptosis

Both ABT-737 and celastrol are reported to modulate apoptosis in cancer cells, thus we are inspired to determine the synergistic effects of ABT-737 plus Celastrol on apoptosis in Bel-7402 and HepG2 cells. Firstly, PI staining for Sub-G1 content analysis was used to characterize apoptosis in Bel-7402 and HepG2 cells treated with 10 µM ABT-737, 1.25 µM Celastrol or the combination for 24 h, 48 h and 72 h. As shown in Fig. 2A, ABT-737 in combination with Celastrol led to more apoptosis than the mono-treatment groups; cells suffering from apoptosis following combination treatment increased time-dependently. The differences of apoptotic cells between combination treatment versus mono-treatment groups were statistically significant in both Bel-7402 and HepG2 cells (*, *p*<0.05; **, *p*<0.01) (Fig. 2A). Typical morphologic features of apoptosis, including chromatin condensation, nuclear fragmentation and formation of apoptotic bodies, were also observed in 10 µM ABT-737, 1.25 µM Celastrol or the combination-treated Bel-7402 and HepG2 cells by DAPI staining. 10 µM ABT-737 in combination with 1.25 µM Celastrol induced more apoptotic bodies than the mono-treatments (Fig. 2B). Caspase-3 is a critical effector caspase in apoptotic pathways, of which the classical substrate is poly(ADP-ribose)polymerase (PARP) [25]. Using western blot analysis, we detected the activation of caspase-3 and cleavage of PARP. In both Bel-7402 and HepG2 cells, although ABT-737 and Celastrol had little effect on caspase-3 and PARP, the combination caused much more significant cleavage of caspase-3 and PARP (Fig. 2C). In HepG2 cells, by introducing the caspase-3 specific substrate Ac-DEVDPNA, ABT-737 and Celastrol co-treatment was demonstrated to trigger a marked increase in caspase-3 activity (4.2-fold compared to control group) (Fig. 2D), whereas the mono-treatment groups did not displayed a evident caspase-3 activation (1.1-fold and 1.0-fold, in Celastrol- and ABT-737-groups, respectively), which was in consistent with the observation in Fig. 2C, further indicating the synergistically apoptosis induced by ABT-737 and Celastrol. Generally, these data demonstrated that the combination of ABT-737 and Celastrol resulted in enhanced apoptosis in Bel-7402 and HepG2 cells.

#### 2.2 Activation of mitochondrial-based apoptotic pathway was triggered by the combination of ABT-737 and celastrol

Next, the mitochondrial membrane potential in the cells exposure to ABT-737, Celastrol or the combination was examined by JC-1 staining. The number of cells shifting from red to green fluorescence indicates the frequency of cells exhibiting mitochondrial depolarization. In 24 h-treated Bel-7402 and HepG2 cells, moderate green fluorescence was observed after the mono-treatments, while green fluorescence replaced most red fluorescence following the combination treatment of ABT-737 and Celastrol (Fig. 2E). Furthermore, we detected the protein levels of Bax, Bcl-2, Bcl-xL, as well as the mitochondrial release of cytochrome c (Fig. 2F and 2G). In both HepG2 and Bel-7402 cells, the accumulation of Bax was detected after treatment of ABT-737 and Celastrol for 24 h, in the contrast, Bcl-2 and Bcl-xL decreased after the combination treatment. We then evaluated the ratio of Bax/Bcl-2 and Bax/Bcl-xL in HepG2 cells, which play important roles in the onset of apoptosis [26]. The combination of ABT-737 and Celastrol significantly upregulated the ratio of Bax/Bcl-2 and Bax/Bcl-xL, increasing from 0.35 to 2.49 (Bax/Bcl-2) and 0.55 to 3.91 (Bax/Bcl-xL), respectivley (Fig. 2F). Additionally, evident release of cytochrome c from mitochondrial to cytoplasm was also induced by ABT-737 plus Celastrol combination (Fig. 2G). Taken together, these results suggested the mitochondrial pathway was involved in the apoptosis triggered by the combination of ABT-737 and Celastrol.

### 3 Kinetics of Noxa Induction Correlated with Activation of Apoptotic Machinery by the Combination of ABT-737 and Celastrol

To characterize the synergistic apoptotic machinery of ABT-737 and Celastrol, western blot analysis was performed on Bel-7402 and HepG2 cells after exposure to 10 µM ABT-737, 1.25 µM Celastrol or the combination for various time intervals. In Bel-7402 cells, kinetic analysis presented that the induction of Noxa proteins became detectible following exposure to 1.25 µM Celastrol or the combination for 3 h. Meanwhile, we noticed that the cleavage of PARP (indicating caspase activation) by the combination became visible after 6 h (Fig. 3A). In HepG2 cells, the induction of Noxa by 1.25 µM Celastrol or the combination, compared with Bel-7402 cells, was delayed with appearance after 24 h, followed by less sensitive PARP cleavage (Fig. 3B). Noticeably, in the two tested cancer cell lines, although the timing of caspase activation and Noxa up-regulation were different from each other, Noxa induction was assuredly observed earlier than the caspase activation, which implied that under the treatment of ABT-737, Celastrol or the combination, Noxa increment was triggered prior to the apoptotic cell death, raising the possibility that Noxa induction played a role in the apoptosis elicited by ABT-737 plus Celastrol.

In addition to Noxa, we noted that the expression of another two BH3-only proteins Bim and PUMA was also upregulated by the combination of ABT-737 and Celastrol (Fig. 3A and 3B), implicating that these two factor may aid in promote the synergistic apoptotic-induction by the combination.

### 4 Noxa was Required in Potent Augmentation of ABT-737-killing by Celastrol

As aforementioned, Noxa probably participate in apoptosis induced by combination treatments of ABT-737 and Celastrol. To identify the involvement of Noxa and its role in the apoptosis, we firstly knocked down Noxa by transfection with Noxa-specific siRNA in HepG2 cells for 48 h. The expression of Noxa was substantially reduced upon transfection with Noxa siRNA (Fig. 4A). Then HepG2 cells transfecting Noxa siRNA were treated with 10 µM ABT-737, 1.25 µM Celastrol or the combination for another 48 h. Fig. 4B showed that Noxa-specific siRNA significantly reduced the cleavage of caspase-3 and PARP by ABT-737 in combination with Celastrol. From three independent experiments, the ratio of cleaved-caspase-3/β-Actin and cleaved-PARP/β-Actin were analyzed (Fig. 4C and 4D), which revealed that knockdown of Noxa evidently resulted in the decrement of the ratios, suggesting the reduced apoptotic potent of ABT-737 plus Celastrol combination in Noxa-silenced cells. Additionally, transfection of Noxa siRNA also cancelled the synergistic anti-proliferative effect by the combination of ABT-737 and Celastrol in HepG2 cells, with the cell survival fraction aggrandizing from 57.14% to 93.56% (Fig. 4E). These data demonstrated that the cooperative anti-cancer effects by ABT-737 and Celastrol required Noxa, of which knockdown attenuated the cytotoxicity and apoptotic effects of ABT-737 plus Celastrol.

### 5 Celastrol Promoted the Binding of Noxa to Mcl-1

The major resistance to ABT-737 is thought to be its low affinity with Mcl-1. Noxa is a BH3-only protein which can efficiently bind to Mcl-1 and inhibit the pro-survival effects of Mcl-1. Given the induction of Noxa by Celastrol and the combination treatment, we determined the interaction between Noxa and its high-affinity partner Mcl-1 that resulted in Noxa/Mcl-1 complexes. To clarify this issue, we immunoprecipitated Mcl-1 protein and probed for Noxa in HepG2 cells treated with 10 µM ABT-737, 1.25 µM Celastrol or the combination for 24 h. As revealed in Fig. 5A, Celastrol mono-treatment enhanced the interaction of Mcl-1 and Noxa in HepG2 cells and Noxa binding to Mcl-1 elevated significantly after the combination treatment; in the meantime, Mcl-1 protein levels dropped in Celastrol-treated and combination-treated HepG2 cells. We further conduct a reverse IP using anti-Noxa antibodies, and also noticed a increment of Mcl-1 bound with Noxa (Fig. 5B). Taken together, induction of Noxa by Celastrol encouraged the interaction of Noxa and Mcl-1, resulted in the reduction of Mcl-1, which might contribute to the enhanced apoptosis induced by ABT-737 and Celastrol combination.

### 6 Celastrol-caused Hsp90 Inhibition and ER-Stress Response Led to the Increase of Noxa

#### 6.1 Celastrol induced degradation of Hsp90 client proteins

In order to elucidate the correlation between the HSP90-targeting effects [27] and the Noxa induction in Celastrol-exposed cells, the degradation of Hsp90 client proteins, such as phospho ERK1/2 and CDK4, was monitored by western blotting in both Bel-7402 and HepG2 cells with serial concentrations of celastrol for 3 h (Fig. 6A and 6B). Additionally, in accordance with previous report, Celastrol also increased Hsp70 expression, which is a hallmark of Hsp90 inhibition [28]. Since Hsp90 client proteins become misfolded and ubiquitinated by Hsp90 inhibition and are then down-regulated by proteasomal degradation [29], we then detected whether Celastrol could induce protein ubiquitination followed by proteasomal degradation. As expected, Celastrol treatment promoted the level of protein ubiquitination (Fig. 6A and 6B). Collectively, these data revealed the inhibition of Hsp90 and degradation of Hsp90 client proteins by Celastrol.

#### 6.2 Celastrol activated eIF2a pathway

ER-stress response is mutually connected with Hsp90 activity, inhibition of which could exert effects on ER stress [30], [31]. Thus we investigated the activation of eIF2α-ATF4 signaling during ER stress. Western blot analysis revealed that upon treatment of Celastrol in HepG2 cells, the phosphorylation of eIF2α would elevated significantly accompanied with increased concentrations of Celastrol; likewise, enhanced expression of ATF4 and Chop protein was consistent with increased phosphorylation of eIF2α under conditions of ER stress elicited by Celastrol (Fig. 6C). Furthermore, ATF4 has been regarded as a transcriptional activator of Noxa [32]. Increased levels of Noxa mRNA were also observed in response to Celastrol or the combination treatment, with the Noxa mRNA induction at 12 h reaching more than 2-fold in the Celastrol group, and achieved around 8-fold in combination group, comparing with control group (Fig. 6D).

#### 6.3 Celastrol-activated eIF2a-ATF4 signaling increased Noxa mRNA level

The up-regulation of ATF4 expression is a potential mediator of Noxa as mentioned above. To test the hypothesis that Celastrol-activated eIF2α-ATF4 signaling resulted in elevated levels of Noxa mRNA, ATF4 expression was down-regulated by specific siRNA in HepG2 cells prior to treatment with Celastrol. ATF4 knockdown was confirmed by western blot analysis (Fig. 6E). As shown in Fig. 6F, while Celastrol increased Noxa mRNA level to 2.76-fold of control group, knockdown of ATF4 resulted in significant inhibition of Noxa mRNA with 1.22-fold of control group (ATF4 siRNA+Celastrol *vs.* control siRNA+Celastrol: *p*<0.05), suggesting that Celastrol-increased Noxa mRNA was ATF4-dependent (Fig. 6F).

## Discussion

Hepatocellular carcinoma (HCC) is one of the most leading causes of cancer-related deaths worldwide. A large number of HCC patients have to be considered for systemic chemotherapeutic treatment, however, the treatment with chemotherapeutic drugs is often ineffective in HCC patients due to the apoptosis resistance, mainly caused by the high level of anti-apoptotic factors such as Mcl-1 [18]. Therefore, more effective treatment regimens for patients with advanced HCC are desperately needed.

ABT-737, a Bad-like BH3 mimetic which selectively bounds to Bcl-2, Bcl-XL and Bcl-w, is highlighted as a promising anti-cancer agent, meanwhile, its low affinity with Mcl-1 and high basal level of Mcl-1 expression in cancer cells is preventing it from being efficient as a single agent. Recently, Hikita et al reported that combining Sorafenib with ABT-737 would efficiently promote apoptosis in HCC cells which were relatively resistant to ABT-737, through the suppression of Mcl-1 by Sorafenib [33]. Together with the observation that ABT-737 synergistically induces apoptosis with overexpression of Noxa that destabilizes or inhibits Mcl-1 [34], we were inspired to explore new treatment regimen(s) taking advantage of the antagonist effect of Noxa to Mcl-1, so as to improve the therapeutic outcome of HCC patients.

Celastrol is a natural compound possessing potent anti-proliferative effects and apoptosis-induction ability in several preclinical cancer models. In a recent study, Celastrol was revealed to induce apoptosis by activating Noxa and modulating Mcl-1 [13]. Consequently, owing to the Noxa-regulating effect of Celastrol, and the Mcl-1-resistant feature of ABT-737, we proposed that the combination of Celastrol with ABT-737 may achieved synergistic anti-cancer activity, and several experiments were performed to prove our hypothesis.

In the current study, ABT-737 and Celastrol were demonstrated to exhibited distinct synergistic anti-cancer effects in HCC cells, as indicated by significant enhanced anti-proliferative activities and apoptosis-induction (Fig. 1, Table 1, Fig. 2). Further studies revealed that Celastrol stimulated high levels of Noxa expression, comparably with that in the co-treatment of ABT-737 and Celastrol group (Fig. 3). Knockdown of Noxa evidently decreased cellular sensitivity to ABT-737 and Celastrol, not only cancelling the co-treatment induced apoptosis (Fig. 4B, 4C and 4D), but the cell growth inhibition as well (Fig. 4E). These results indicated that the upregulation of Noxa was indispensable for ABT-737-plus-Celastrol-induced HCC cell death. In accordance with the high affinity of Noxa with Mcl-1, as Celastrol elevated the level of Noxa protein, a greater extent of Noxa increment was observed in IP samples treated with anti-Mcl-1 antibodies in Celastrol and combination groups (Fig. 5A, Fig. S1, IP-Noxa *vs.* WB Noxa), suggesting a remarkable interaction between Noxa and Mcl-1 promoted by Celastrol or the combination. Reverse IP displayed similar results (Fig. 5B), further supporting the enhanced bound between Noxa and Mcl-1. Upon Celastrol treatment, either alone or with ABT-737, Mcl-1 levels were reduced (Fig. 5A), probably owing to the increased interaction with Noxa and the subsequent proteasomal degradation of Mcl-1 [35]. Thus, one of the molecular mechanisms for the synergy between ABT-737 and Celastrol is that ABT-737 neutralized Bcl-2, Bcl-XL, Bcl-w, whereas Celastrol induced Noxa levels, inactivating the anti-apoptotic function of Mcl-1. Together, our data indicated that Noxa increment and its binding to Mcl-1 are critical determinants for the synergism of ABT-737 and Celastrol.

Mechanistically, proteasome and HSP90 have been shown to be the key targets of Celastrol [15]–[17], [36]. However, by acting on which of them, Celastrol was conferred to possess the ability to induce Noxa mRNA and protein level, is remained to be elucidated. As mentioned above, Celastrol increased the protein expression of Noxa [13], with detailed mechanisms remain elusive. The author proposed that in their models, Celastrol induced Noxa activation in a p53-independet manner, probably through its proteasome-inhibitory effects, since it has been reported that although Noxa is not a direct substrate of proteasome [37], its mRNA is transcriptionally enhanced by a proteasome inhibitor [37], [38].

In our study, in an attempt to understand the detailed pathways that might be regulating Noxa upon Celastrol exposure, we noticed the activation of eIF2α-ATF4 signaling pathway, which is one of the essential pathways during ER stress response. Our results revealed that the phosphorylation of eIF2α and expression of ATF4 protein were obviously elevated in a concentration-dependent manner in Celastrol-treated cells under conditions of ER stress (Fig. 6C), accompanied with enhanced ubiqutination as well as elevated Noxa protein and mRNA levels (Fig. 6D). The finding that ER stress was activated upon Celastrol treatment were supported by the previous reports that Celastrol also acts as HSP90 inhibitor which could induce the activation of ER stress [15]–[17], and was further confirmed by our data that the splicing of XBP-1 which denoted the activation of IRE1-mediated ER stress (Fig. S2). Importantly, by knocking-down ATF4 expression using ATF4-specific siRNA (Fig. 6E), Noxa mRNA activation was greatly attenuated, indicating the essential role of ATF4 in Celastrol-induced elevation of Noxa mRNA (Fig. 6F). The ATF4-dependent induction of Noxa by Celastrol revealed in this study was in accordance with the report regarding ATF4 as a transcriptional activator of Noxa [39]. Together with previous report, we were encouraged to disclose that the induction of ER stress and ATF4 by Celastrol played critical roles for the upregulation of Noxa. Unexpectedly, although the Noxa induction was abolished, the apoptosis triggered by the combination could only be partially attenuated by ATF4 siRNA (Fig. S3). As a widely-regarded stress-responsive gene, ATF4 not only activates pro-apoptotic effects, but also plays a protective role by regulating cellular adaptation to adverse conditions, by regulating a wide range of downstream target genes [22]. Consequently, the silence of ATF4 may result in aggrandized cell death, which counteracted the cell survival mediated by the Noxa reduction. Based on these findings, we speculated that those ER stress-activating agents may increase the expression of Noxa, leading to the reduction or inhibition of Mcl-1; and this characteristic may merit them as potential combination candidates with ABT-737 by exerting synergistic anti-cancer activities.

In conclusion, our findings indicated that through the inhibition of Hsp90, and the induction of ER stress, specifically, the activation of eIF2α-ATF4 pathway, Celastrol promoted Noxa upregulation and its binding to Mcl-1, consequently, abrogated the anti-apoptotic effects of Mcl-1 which was the major resistance factor for ABT-737. Thus, the combination of ABT-737 and Celastrol synergistically suppressed HCC cell proliferation and induced these cells to undergo apoptosis. These results not only illuminated potential strategy to enhance the therapeutic efficacy of ABT-737 by co-treating with Celastrol, but also implicated the synergy of these two agent was attributed to, at least partially, the activated ER stress response and the subsequent upregulation of Noxa triggered by Celastrol; additionally, the current findings also gave new insights into the mechanisms by which Celastrol caused elevated Noxa expression in cancer cells on one hand, and sufficiently understanding the mechanisms forms the crucial basis for the effective combination of Celastrol with those agents acting on apoptosis pathways in potential clinical settings on the other.

## Supporting Information

Figure S1 **The protein density analyses of** **Figure 5A** **.** IP-Noxa: Noxa bands from the samples treated with anti-Mcl-1 antibodies; WB-Noxa: Noxa bands from total cell lysates. Normalized to the Noxa density of untreated cells for each group.
(TIF)

Figure S2 **The ER stress caused by Celastrol. A.** Celastrol caused XBP-1 splicing in HepG2 cells when treated for 3 h. **B.** ATF4 basal expression in HepG2 cells remained unchanged.
(TIF)

Figure S3 **ATF4 siRNA partially attenuated the apoptosis by Celastrol and ABT-737.** HepG2 cells were transfected with ATF4A siRNA according to manufacturer’s recommendations. Forty-eight hours after transfection, cells were treated with 10 µM ABT-737, 1.25 µM Celastrol and the combination for 48 h. Lysates were harvested and immunobloted with caspase-3 antibody.
(TIF)
