# Supplementary figures and images for: Upregulating Noxa by ER Stress, Celastrol Exerts Synergistic Anti-Cancer Activity in Combination with ABT-737 in Human Hepatocellular Carcinoma Cells
Source: PLoS One. 2012 Dec 20;7(12):e52333. doi: 10.1371/journal.pone.0052333 (PMC3527540; doi:10.1371/journal.pone.0052333)

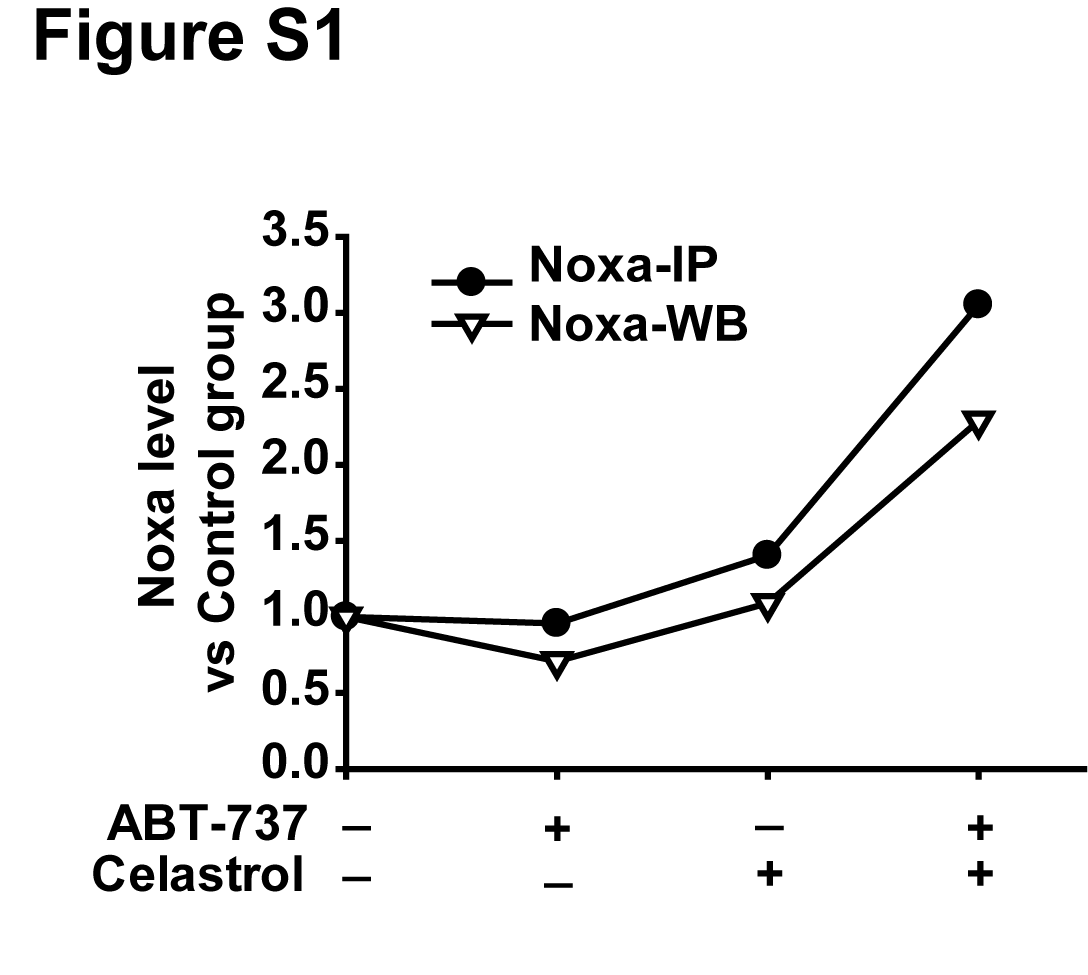

Supplement: Figure S1 — The protein density analyses of Figure 5A . IP-Noxa: Noxa bands from the samples treated with anti-Mcl-1 antibodies; WB-Noxa: Noxa bands from total cell lysates. Normalized to the Noxa density of untreated cells for each group. (TIF) [file pone.0052333.s001.tif]

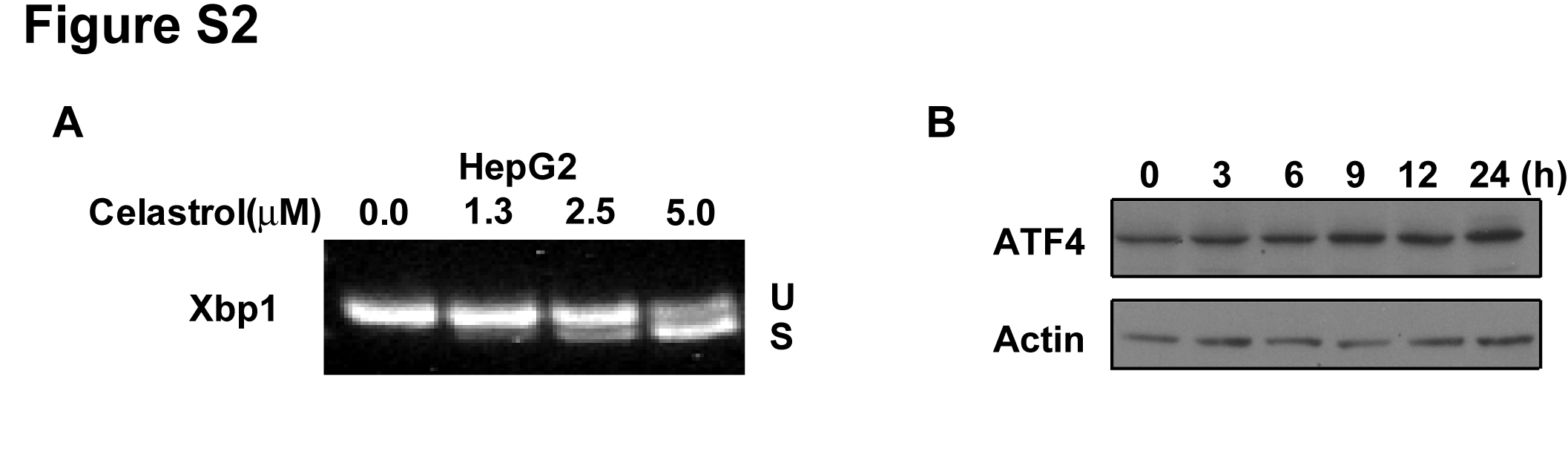

Supplement: Figure S2 — The ER stress caused by Celastrol. A. Celastrol caused XBP-1 splicing in HepG2 cells when treated for 3 h. B. ATF4 basal expression in HepG2 cells remained unchanged. (TIF) [file pone.0052333.s002.tif]

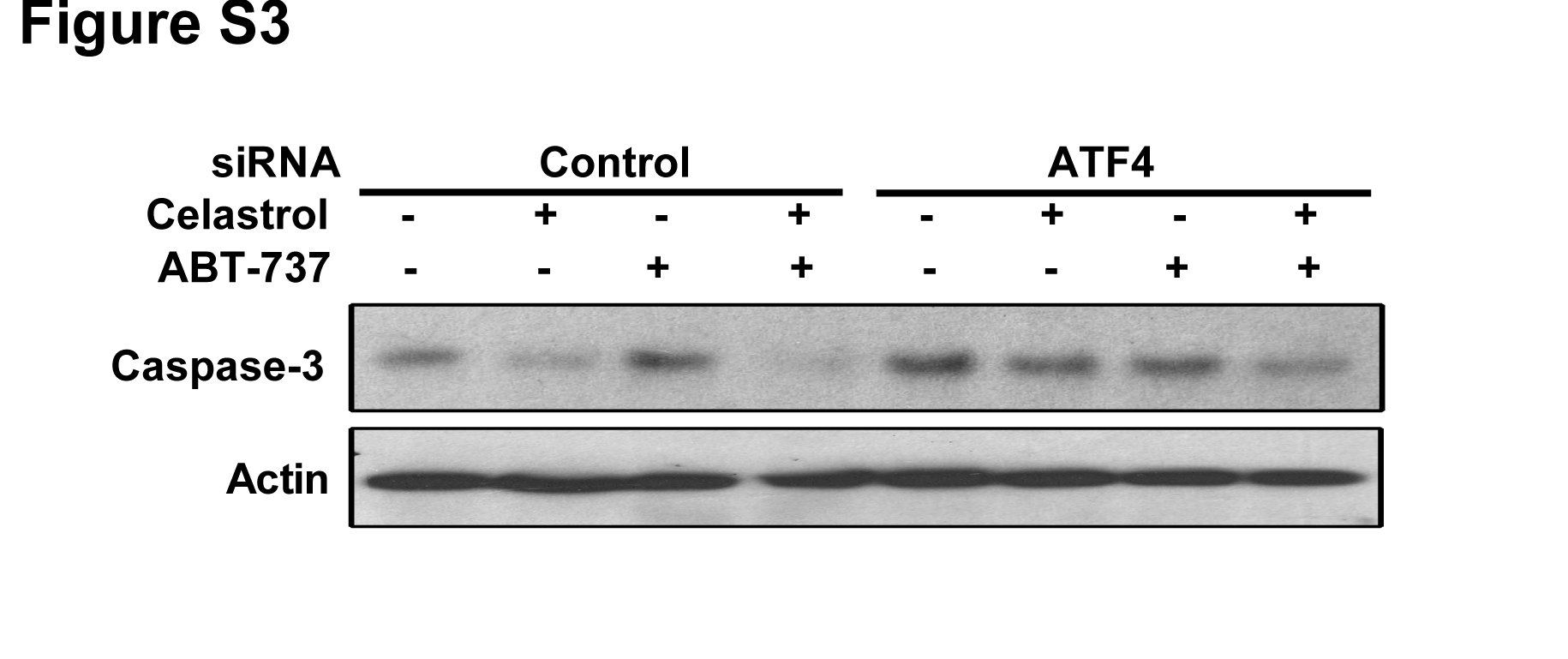

Supplement: Figure S3 — ATF4 siRNA partially attenuated the apoptosis by Celastrol and ABT-737. HepG2 cells were transfected with ATF4A siRNA according to manufacturer’s recommendations. Forty-eight hours after transfection, cells were treated with 10 µM ABT-737, 1.25 µM Celastrol and the combination for 48 h. Lysates were harvested and immunobloted with caspase-3 antibody. (TIF) [file pone.0052333.s003.tif]
